# Effects of Redox Modulation on Cell Proliferation, Viability, and Migration in Cultured Rat and Human Tendon Progenitor Cells

**DOI:** 10.1155/2017/8785042

**Published:** 2017-07-02

**Authors:** Yuk Wa Lee, Sai Chuen Fu, Man Yi Yeung, Chun Man Lawrence Lau, Kai Ming Chan, Leung Kim Hung

**Affiliations:** ^1^Department of Orthopaedics and Traumatology, Faculty of Medicine, The Chinese University of Hong Kong, Shatin, Hong Kong; ^2^Department of Orthopaedics and Traumatology, Prince of Wales Hospital, Shatin, Hong Kong

## Abstract

Tendon healing is slow and usually results in inferior fibrotic tissue formation. Recently, application of tendon derived stem cells (TDSCs) improved tendon healing in animal studies. In a chicken model, local injection of antioxidants reduced tendon adhesion after tendon injury. An in vitro study demonstrated that supplementation of H_2_O_2_ reduced tenogenic marker expression in TDSCs. These findings suggested that the possibility of TDSCs is involved in tendon healing and the cellular activities of TDSCs might be affected by oxidative stress of the local environment. After tendon injury, oxidative stress is increased. Redox modulation might affect healing outcomes via affecting cellular activities in TDSCs. To study the effect of oxidative stress on TDSCs, the cellular activities of rat/human TDSCs were measured under different dosages of vitamin C or H_2_O_2_ in this study. Lower dose of vitamin C increased cell proliferation, viability and migration; H_2_O_2_ affected colony formation and suppressed cell migration, cell viability, apoptosis, and proliferation. Consistent with previous studies, oxidative stresses (H_2_O_2_) affect both recruitment and survival of TDSCs, while the antioxidant vitamin C may exert beneficial effects at low doses. In conclusion, redox modulation affected cellular activities of TDSCs and might be a potential strategy for tendon healing treatment.

## 1. Introduction

Tendon injuries, both acute ruptures and chronic degeneration, are a common clinical problem. Tendon healing is a slow process and commonly results in inferior fibrotic tissue that increases the risk of reinjury [1]. In order to promote tendon healing, various approaches to enhance regeneration of tendinous tissues instead of fibrous tissues are developed [2]. Recently, tendon progenitor cells (TPC) with pluripotency were identified as one of the cell sources of tendon healing [3, 4] and attempted to use exogenous tendon-derived stem cells (TDSCs) to promote tendon healing are under active investigation [5]. Obviously, it would be simpler to mobilize endogenous TDSCs, but the processes of recruitment of TDSCs in situ are still unclear.

Recruitment of healing cells occurs at the late inflammatory phase and proliferation phase of healing process. The process primarily involves activation of progenitor cells and migration of activated cells to the wound, while the amounts of recruited cells would also be determined by cell viability, cell proliferation, and programmed cell death. It is well-known that cell recruitment to wound is affected by changes in local biochemical environment after injuries, such as cytokines [2, 6] and oxidative stress [7]. Our previous study showed that antagonizing posttraumatic oxidative stress by vitamin C [8] or vitamin E [9] was able to reduce fibrotic tendon adhesion in a chicken model. On the other hand, cell proliferation of TDSCs, as well as the gene expression of tenomodulin and elastin, was significantly suppressed by H_2_O_2_ treatment [10]. It is possible that recruitment of TDSCs is affected by the redox status in the wound, which may in turn affect the healing outcome. In this study, we propose to investigate the effects of redox modulation on recruitment of tendon cells in vitro. Cell recruitment was investigated by measurement of clonogenicity of cultured TDSCs, as well as cell migration, proliferation, viability, and programmed cell death of TDSCs at passages 3–5 in both rat TDSCs and human TDSCs. The effects of different doses of vitamin C or hydrogen peroxide on these cellular activities were investigated.

## 2. Materials and Methods

### 2.1. Primary Cell Culture of Human and Rat TDSCs

Collection of human samples was approved by the Clinical Research Ethics Committee (CREC reference number 2013.479), the Chinese University of Hong Kong. Human hamstring tendons were collected from three patients undergoing anterior cruciate ligament reconstruction using hamstring autografts after obtaining their consent. Residual tissues from the hamstring tendon autografts were collected. Cares were taken to avoid contamination from other connective tissues. Animal ethical approval was obtained for preparation of rat TDSCs with a similar protocol (10/010/DRG). Rat patellar tendons were harvested for preparation of TDSCs.

Isolation of TDSCs was done following established protocol [4, 11]. Briefly, care was taken that peritendinous connective tissue was carefully removed. Tendon was washed in sterile phosphate-buffered saline (PBS) and cut into small pieces. Rat patellar tendon tissue was further digested with type I collagenase (3 mg/mL, Sigma-Aldrich) for 2 hours, while human hamstring tendon was further digested with type I collagenase (3 mg/mL) with 1 U of dispase for 3.5 hours at 37°C. To remove the debris and collagenase after collagenase digestion, tendon tissue passed through a 70 *μ*m cell strainer (Becton Dickinson, Franklin Lakes, NJ) to yield a single nucleated cell suspension. The released cells were washed with PBS and resuspended in a complete culture medium (low-glucose Dulbecco's Modified Eagle's Medium (LG-DMEM, Gibco)), 10% heated inactivated fetal bovine serum, 50 *μ*g/mL penicillin, 50 *μ*g/mL streptomycin, and 100 *μ*g/mL neomycin (all from Invitrogen, Carlsbad, CA, USA). The isolated nucleated cells were seeded at an optimized low density (500 cells/cm^2^) and cultured at 37°C, 5% CO_2_. At day 3, cells were washed with PBS and medium was changed to remove all the nonadherent cells. At days 7–10, the adherent cells were trypsinized and mixed together as passage 0 (P0). Medium was changed every three days. TDSCs were subcultured when they reached 80–90% confluence. All experiments were performed at passage 3 unless stated otherwise.

### 2.2. Colony-Forming Assay

Human or rat TDSCs were seeded at 50 cells/cm^2^ in control medium or test media. Based on our pilot studies, 100 (LD), 200 (MD), 500 (HD) *μ*M H_2_O_2_, or vitamin C were used to test the treatment effects (*n* = 6). The cells were cultured for 7 days, fixed with ethanol, and stained with 0.5% crystal violet for counting of colony-forming units (CFU). CFU-t and CFU-f were defined as CFU of tendon-like cell and CFU of fibroblast-like cell. CFU were counted with the help of ImagePro Premiere 9.2 software by thresholding the staining by crystal violet. Circular area of interest (AOI) at 1 mm diameter was used to define countable CFU according to staining inside AOI, with less than 15% area filled with cells were not counted.

### 2.3. Cell Migration Assay

Chemotaxis was assayed using the Transwell system with 8.0 *μ*m pore polycarbonate membrane (Corning). 1 × 10^5^ cells were seeded in the insert well and incubated with serum-free media. After 24 hours of preincubation, serum free-media in the lower compartment were replaced with [1] serum-free medium to serve as baseline control for random movement [2], medium with 10% serum to serve as positive control of chemotaxis, or [3] medium with 10% serum and H_2_O_2_ or vitamin C at the test doses. After 24 hours of incubation with these testing media (*n* = 3), the insert wells were washed and membrane was fixed with 4% formaldehyde. Cells remaining on the membrane facing to the side of insert well were mechanically removed with cotton swabs. Cells passing through the membrane were counted under microscope after DAPI staining. Directional cell migration is differentiated from random cell movement by setting up controls containing 10% FBS in the insert wells.

### 2.4. Alamar Blue and BrdU Assays

At P2-3, TDSCs were seeded (4 × 10^3^ cells/cm^2^). When it reaches subconfluence, culture medium was replaced with control medium or test media. Cell viability was evaluated using the Alamar Blue assay according to the manufacture's protocol (Serotec, catalog number BUF012A). TDSCs were seeded in 96-well cell culture plates in 6000 cells/well. After 24 hours incubation of various culture mediums, Alamar Blue solution was diluted with different culture media to a final concentration of 10% Alamar Blue solution. After 3 hours of Alamar Blue solution incubation, all plates were measured at 570 nm and 600 nm.

Cell proliferation was determined by bromodeoxyuridine (BrdU) assay (cell proliferation ELISA, BrdU (colorimetric), Roche) as according to the manufacture's protocol. TDSCs were seeded in 96-well cell culture plates in 6000 cells/well. After cell reached confluence, cells were treated with different culture media for 24 hours before BrdU assay.

### 2.5. Caspase Assay

Cell apoptotic activity was determined by Caspase-Glo® 3/7 Assay (Promega, catalog number G8090) according to the manufacture's protocol. In brief, TDSCs at passage 3 were seeded in 96-well cell culture plates in 6000 cells/well. After cells reached confluence, cells were treated with different cell culture media for 24 hours. Caspase assay was done afterward. Apoptosis is assayed by the caspase 3/7 activity kit.

### 2.6. Statistical Methods

The CFU assay, cell migration assay, cell viability, proliferation, and apoptosis were shown in boxplots. Nonparametric Kruskal-Wallis test was used to perform multiple group comparisons, and *p* values are reported. Two-group comparisons were performed with the Mann–Whitney *U* test with the Bonferroni correction when necessary (*a* = 0.05/2 = 0.025). All the data analysis was done using the Statistical Package for the Social Sciences (SPSS) 21 (IBM, Armonk, New York). *p* ≤ 0.050 was regarded as statistically significant.

## 3. Results

### 3.1. Colony-Forming Assay

**Figure 1:**
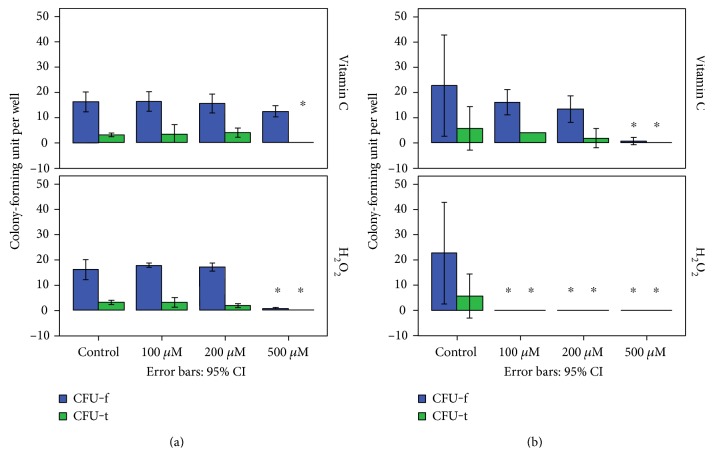
Effect of redox modulation on rat (a)/human (b) TDSCs on CFU assay. CFU-t is tendon cell-like colony; CFU-f is fibroblast cell-like colony; ∗ indicates that the *p* value is <0.05 as compared with the control group (Mann–Whitney *U* test).

In rat TDSCs, the total number of CFU was significantly decreased by incubating with 500 *μ*M vitamin C, and it was mainly due to suppression of CFU-t (*p* = 0.002), but CFU-f was not affected (*p* = 0.089). Incubation with 500 *μ*M H_2_O_2_ abolished formation of CFU-t and extensively suppressed CFU-f. In human TDSCs, vitamin C from 100 to 500 *μ*M exhibited a dose-dependent decrease in total number of CFU, while no observable CFU was found in all H_2_O_2_-treated groups. Both CFU-t and CFU-f were affected to the same extent by vitamin C in human TDSCs (Figure 1).

### 3.2. Cell Migration Assay

**Figure 2:**
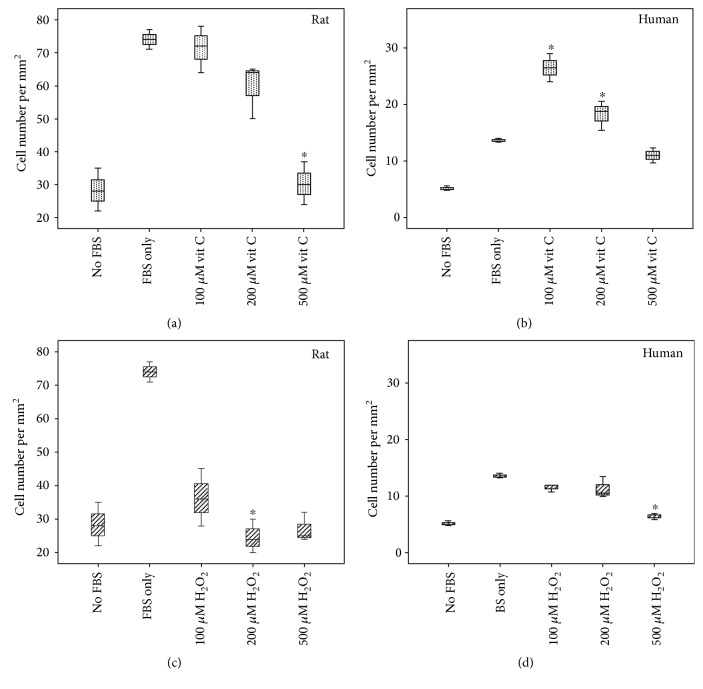
Boxplots show the effect of redox modulation on rat (a, c)/human (b, d) TDSCs on cell migration. In (a, b), cells were treated with vitamin C; in (c, d), cells were treated with H_2_O_2_. ∗ indicates that the *p* value is <0.05 as compared with the FBS-only group (Mann–Whitney *U* test).

In rat TDSCs, vitamin C exhibited a dose-dependent suppression of cell migration and 500 *μ*M vitamin C abolished the directional migration induced by FBS, while H_2_O_2_ at all test doses abolished cell migration (Figures 2(a) and 2(c)). In contrast, vitamin C enhanced TDSC cell migration in human TDSCs at lower doses (100 and 200 mM); a dose-dependent suppression in cell migration was also observed in the H_2_O_2_-treated human TDSCs (Figures 2(b) and 2(d)).

### 3.3. Viability, Proliferation, and Apoptosis

**Figure 3:**
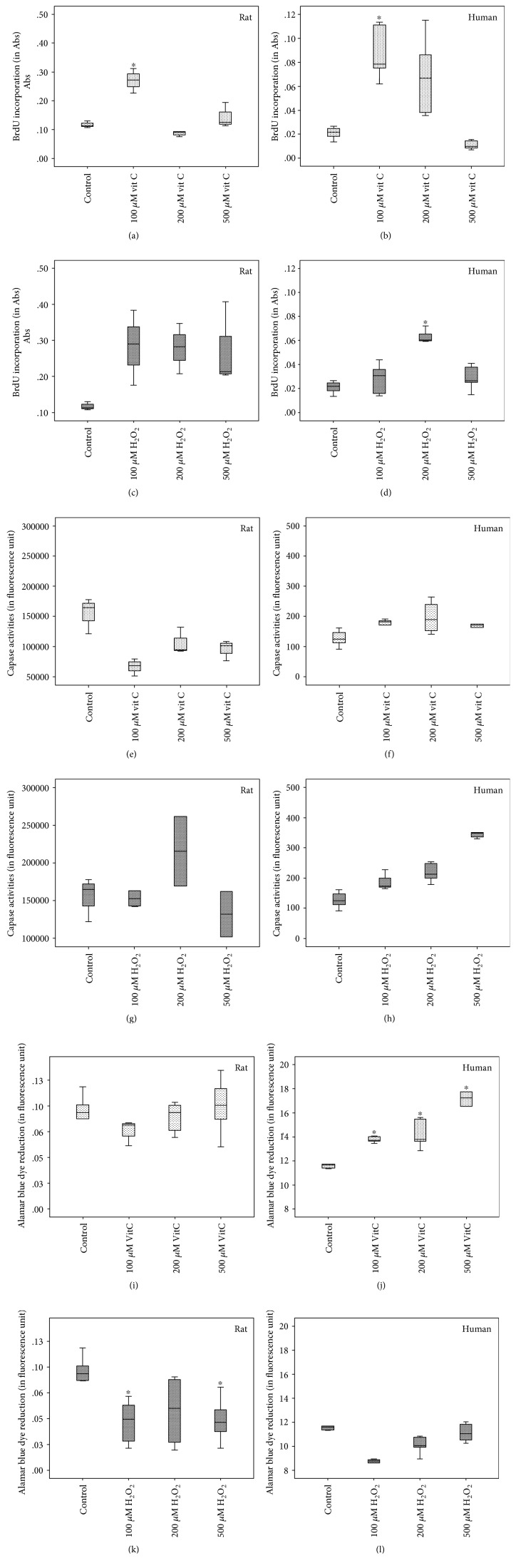
Boxplots show the effect of redox modulation on rat (a, c, e, g, i, k)/human (b, d, f, h, j, l) TDSCs on cell proliferation (a, c, b, d), apoptosis (e, g, f, h), and cell viability (i, k, j, l). In (a, e, i, b, f, j), cells were treated with vitamin C; in (c, g, k, d, h, l), cells were treated with H_2_O_2_. ∗ indicates that the *p* value is < 0.05 as compared with the FBS-only group (Mann–Whitney *U* test).

In rat TDSCs, cell proliferation was enhanced at a low dose of vitamin C (Figure 3(a)), but no significant change in cell viability and apoptosis was found in all test doses of vitamin C (Figures 3(i) and 3(c)). H_2_O_2_ decreased cell viability at all test doses in rat TDSCs (Figure 3(f)). In human TDSCs, vitamin C also enhanced cell proliferation at lower doses (*p* = 0.009) (Figure 3(b)), and a dose-dependent increase in cell viability was observed (Figure 3(j)). No change in apoptosis was detected under all test doses of vitamin C (Figure 3(f)). In contrast, H_2_O_2_ increased apoptosis in a dose-dependent manner (Figure 3(h)), but it only stimulated proliferation at 200 *μ*M (Figure 3(d)) and decreased cell viability at 100 *μ*M in human TDSCs (Figure 3(l)).

## 4. Discussion

A summary to compare the effects of redox modulation on human and rat TDSCs is shown in Table 1. Our results showed that hydrogen peroxide exerted similar effects on human and rat TDSCs. In general, hydrogen peroxide promoted apoptosis, suppressed cell viability and cell migration, and inhibited clonogenicity. These findings support that oxidative stress is deleterious to cell recruitment for tendon healing, which explains why antioxidant supplementation at early healing stages is beneficial, as recruitment of progenitor cells for healing is safeguarded. In contrast to H_2_O_2_, the effects of vitamin C exhibited marked differences between human and rat TDSCs. Vitamin C promoted cell migration and cell viability in human TDSCs but not in rat TDSCs. These discrepancies may be rooted from the fact that rats can synthesize vitamin C from glucose in the liver, while humans cannot. As the reserves of vitamin C in human tissues are considerably lower than those in rat [12, 13], human tissues are more sensitive to varying levels of vitamin C. It may explain why human TDSCs were more sensitive to vitamin C supplementation with respect to cell migration and viability. It is speculated that the effects of vitamin C supplementation to promote tendon/ligament healing may be more pronounced in humans. Similar to our observation, in a canine mesenchymal stem cell study, Quintanilha et al. reported that cMSCs overcame thioacetamide-induced oxidative stress in vitro [14]. The antioxidant properties of cMSCs might have overcome oxidative stress because canine can synthesize vitamin C. In our previous tendon adhesion chicken studies, local injection of antioxidants after flexor digitorum profundus tendon injury improved tendon healing. Chicken do not have vitamin C synthesis ability; further study on chicken cells might provide more insight on the difference in cellular activity of stem cell under oxidative stress in relation to the vitamin C synthesis ability.

Previous animal studies showed that the beneficial effects of vitamin C supplementation to promote healing for tendon [8] and ligament injuries [15] were only observed at low dose of vitamin C, but not at a higher dose. Similar results were observed in the current in vitro studies: cell proliferation was promoted by low dose vitamin C, but inhibitory effects on cell migration were observed at higher doses of vitamin C. Low levels of ROS are found to promote MSC proliferation and migration by activating the extracellular signal-regulated kinases (ERK) 1/2 and Jun-1/2 pathways and mediated by the NOXs family. High doses of vitamin C may scavenge too much ROS and exert negative impacts on cell recruitment [16–19]. It may help to explain the biphasic response of vitamin C on cell recruitment for tendon healing.

The major limitation of the present study is the lack of measurement of antioxidants and antioxidant enzymes in the cultured TDSCs, which may affect their sensitivity to redox modulation in vitro. Moreover, the molecular pathways responsible for the redox modulation were not investigated. Putatively, all redox sensitive signaling pathways would be affected, such as NF-kB pathways and peroxiredoxin pathways. It has been suggested that oxidative stress plays a role in the pathogenesis of tendinopathy, with the findings that peroxiredoxin V expression was increased in tendinopathic tissues [20] and that tendon cells were particularly vulnerable to prooxidants like fluoroquinolones [21] which was reported to cause spontaneous tendon ruptures. As shown in the current studies, oxidative stress may negatively affect tendon healing by suppressing TDSC recruitment. Further investigation of redox modulation on tendon cells may help to explore the mechanism of failed healing in tendinopathy.

## 5. Conclusion

Oxidative stress affects both recruitment and survival of tendon progenitor cells, while antioxidants may exert beneficial effects at low doses.

## Figures and Tables

**Table 1:** Summary of the effects of redox modulation on rat/human TDSCs. LD = 100 *μ*M, MD = 200 *μ*M, HD = 500 *μ*M.

		Rat TDSCs	Human TDSCs
CFU	Vitamin C	↓	↓
	H_2_O_2_	↓HD	↓
Migration	Vitamin C	↓	↑
	H_2_O_2_	↓	↓
Proliferation	Vitamin C	↑LD	↑LD
	H_2_O_2_	—	↑MD
Apoptosis	Vitamin C	—	—
	H_2_O_2_	—	↑
Viability	Vitamin C	—	↑
	H_2_O_2_	↓	↓
